# Modelling amyotrophic lateral sclerosis: progress and possibilities

**DOI:** 10.1242/dmm.029058

**Published:** 2017-05-01

**Authors:** Philip Van Damme, Wim Robberecht, Ludo Van Den Bosch

**Affiliations:** 1 KU Leuven, University of Leuven, Department of Neurosciences, Experimental Neurology, andLeuven Research Institute for Neuroscience and Disease (LIND), B-3000 Leuven, Belgium; 2 VIB – Center of Brain & Disease Research, Laboratory of Neurobiology, B-3000 Leuven, Belgium; 3 University Hospitals Leuven, Department of Neurology, B-3000 Leuven, Belgium

**Keywords:** *C. elegans*, Fruit fly, Motor neuron, Neurodegeneration, Zebrafish, iPSCs

## Abstract

Amyotrophic lateral sclerosis (ALS) is a neurodegenerative disorder that primarily affects the motor system and presents with progressive muscle weakness. Most patients survive for only 2-5 years after disease onset, often due to failure of the respiratory muscles. ALS is a familial disease in ∼10% of patients, with the remaining 90% developing sporadic ALS. Over the past decade, major advances have been made in our understanding of the genetics and neuropathology of ALS. To date, around 20 genes are associated with ALS, with the most common causes of typical ALS associated with mutations in *SOD1*, *TARDBP*, *FUS* and *C9orf72*. Advances in our understanding of the genetic basis of ALS have led to the creation of different models of this disease. The molecular pathways that have emerged from these systems are more heterogeneous than previously anticipated, ranging from protein aggregation and defects in multiple key cellular processes in neurons, to dysfunction of surrounding non-neuronal cells. Here, we review the different model systems used to study ALS and discuss how they have contributed to our current knowledge of ALS disease mechanisms. A better understanding of emerging disease pathways, the detrimental effects of the various gene mutations and the causes underlying motor neuron denegation in sporadic ALS will accelerate progress in the development of novel treatments.

## Abstract (precis)

**Summary:** In this Review, Ludo Van Den Bosch and colleagues discuss the different model systems for studying ALS and how they have contributed to our current understanding of the etiology and pathology of this neurodegenerative disease.

## Introduction

Amyotrophic lateral sclerosis (ALS) is a neurodegenerative disorder that primarily affects the motor neurons (MNs) in the motor cortex, brainstem and spinal cord, resulting in progressive muscle weakness (Rowland and Shneider, 2001). It usually has a focal onset, presenting with unilateral limb weakness or with bulbar (Box 1) dysfunction, and it has a tendency to propagate within the motor system network. In the Western world, ALS has an incidence rate of 1-2 individuals per 100,000 per year and a prevalence of 4-8 per 100,000 (Logroscino et al., 2010). The lifetime risk of developing ALS is estimated to be 1 in 400 (Johnston et al., 2006). In ∼10% of ALS patients, the disease runs in the family (familial ALS). The remaining 90% of patients are classified as having sporadic ALS, although causative mutations have been identified in 5-10% of cases (Al-Chalabi et al., 2017; Debray et al., 2013).

ALS usually presents with muscle weakness in limb muscles in ∼2 out of 3 patients or in bulbar muscles in around 1 out of 3 patients (Rowland and Shneider, 2001). Rare disease presentations include weakness in respiratory or axial muscles (Swinnen and Robberecht, 2014). Muscle weakness is typically accompanied by hyper-reflexia, the wasting of muscles and the occurrence of fasciculations due to combined upper and lower motor neuron involvement (Box 1). In about 50% of cases, the degenerative process extends to the frontal and anterior temporal lobe, giving rise to a variable degree of executive dysfunction (Box 1), language impairments or behavioural changes. Approximately 10% of affected individuals develop frontotemporal dementia (FTD, Box 1).

ALS is diagnosed clinically, based on the recognition of both upper motor neuron symptoms (including hyper-reflexia, slowing of fast movements and increased muscle tone) and lower motor neuron signs (such as fasciculations and muscle wasting), in the presence of a progressively worsening disease and in the absence of other pathologies that could explain symptoms. ALS causes relentlessly progressive muscle weakness in most patients, and its effects on respiratory muscles limit survival to 2-5 years after disease onset. The only approved drug for ALS is riluzole, which has a limited but significant effect on patient survival (Bensimon et al., 1994). Although riluzole has different mechanisms of action, its protective effects in ALS are believed to be mediated by the inhibition of glutamate release, counteracting excitotoxicity (Box 2) (Doble, 1996). However, the standard treatment for ALS remains multidisciplinary care, including nutritional and respiratory support and symptom management.

**Fig. 1.**
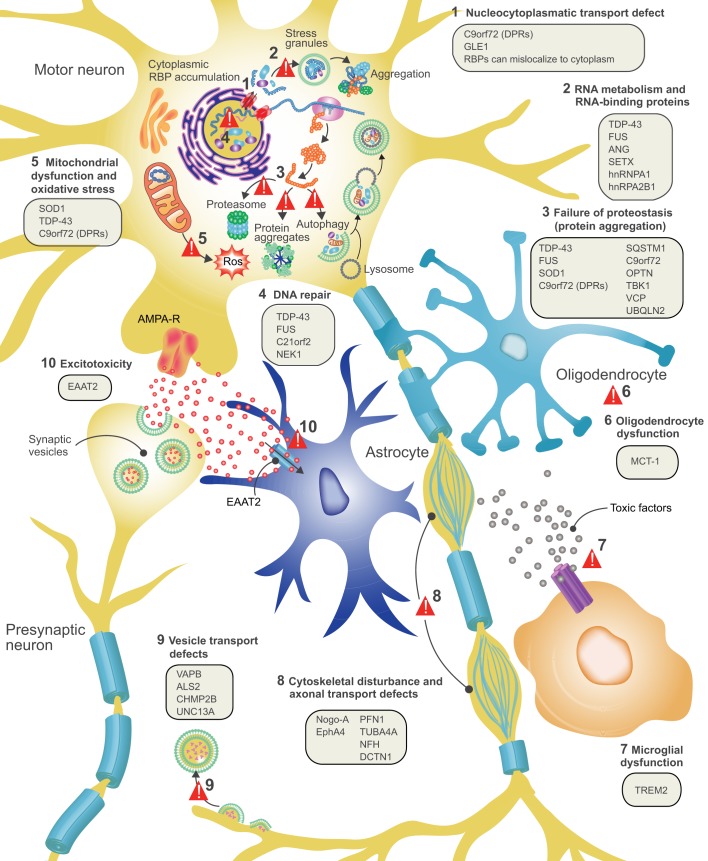
**ALS disease pathology and proposed disease mechanisms.** At the level of cell pathology, ALS is characterized by axonal retraction and cell body loss of upper and lower motor neurons, surrounded by astrogliosis and microgliosis (see Box 2), with ubiquitin- and p62-positive inclusions in surviving neurons. Proposed disease mechanisms contributing to motor neuron degeneration are: (1) Alterations in nucleocytoplasmic transport of RNA molecules and RNA-binding proteins. (2) Altered RNA metabolism: several important RNA-binding proteins become mislocalized in ALS, with cytosolic accumulation and nuclear depletion. The nuclear depletion causes defects in transcription and splicing. Some RNA-binding proteins can undergo liquid-liquid phase separation and can be recruited to stress granules (TDP-43, FUS, ATXN2, hnRNPA1/A2). Altered dynamics of stress granule formation or disassembly can propagate cytoplasmic aggregate formation. (3) Impaired proteostasis with accumulation of aggregating proteins (TDP-43, FUS, SOD1, DPRs). Overload of the proteasome system and reduced autophagy may contribute and/or cause this accumulation. (4) Impaired DNA repair: two recently identified ALS genes (see main text for details) work together in DNA repair, suggesting that impaired DNA repair could also contribute to ALS pathogenesis. (5) Mitochondrial dysfunction and oxidative stress: several ALS-related proteins (SOD1, TDP-43, C9orf72) can enter mitochondria and disrupt normal functioning, with increased formation of reactive oxygen species (ROS) as a consequence. (6) Oligodendrocyte dysfunction and degeneration, leading to reduced support for motor neurons. (7) Neuroinflammation: activated astrocytes and microglia secrete fewer neuroprotective factors and more toxic factors. (8) Defective axonal transport: several ALS-related mutations cause disorganization of the cytoskeletal proteins and disrupt axonal transport. (9) Defective vesicular transport: several ALS-related proteins (VABP, ALS2, CHMP2B, UNC13A) are involved in vesicular transport, suggesting that impaired vesicular transport contributes to ALS pathogenesis. (10) Excitotoxicity: loss of the astroglial glutamate transporter EAAT2 causes accumulation of extracellular glutamate, which causes excessive stimulation of glutamate receptors (e.g. AMPA receptors) and excessive calcium influx.

Disease-causing gene mutations have been identified in ∼80% of patients with familial ALS, and these mutations are typically inherited in an autosomal dominant manner (Al-Chalabi et al., 2017). These mutations are also present in 5-10% of patients with sporadic (non-familial) ALS (Al-Chalabi et al., 2017; Debray et al., 2013); however, the basis of ALS remains unknown in the majority of cases. Over 20 genes have been linked to ALS so far, with more expected in the future (Al-Chalabi et al., 2012). The most common ALS disease-causing gene mutations are found in: *C9orf72* (which encodes chromosome 9 open reading frame 72), it is responsible for 10-15% of all ALS and contains a hexa nucleotide repeat expansion; *SOD1* (superoxide dismutase 1), responsible for 2% of ALS; *TARDBP* (TAR DNA-binding protein 43), responsible for 0.9% of ALS; and *FUS* (fused in sarcoma), responsible for 0.7% of ALS (Al-Chalabi et al., 2017; Renton et al., 2014). Many other less-frequent genetic mutations have also been associated with ALS, including mutations in *UBQLN2* (ubiquilin 2), *OPTN* (optineurin), *VCP* (valosin-containing protein) and *TBK1* (TANK-binding kinase 1) (Al-Chalabi et al., 2017; Renton et al., 2014). At the cellular level, ALS pathology is characterized by axonal retraction, loss of cell bodies of the upper and lower motor neurons, occurrence of astrogliosis and microgliosis, and by ubiquitin-positive inclusions in surviving neurons (Fig. 1) (Saberi et al., 2015). In ∼95% of patients, these inclusions are positive for the RNA-binding protein TDP-43. TDP-43 pathology is found in most ALS patients, and rare mutations in its gene (*TARDBP*) can cause ALS. It is therefore considered a key disease protein in ALS (Saberi et al., 2015).

The clinical manifestation of ALS is also highly variable, in terms of its age at onset, site of onset, disease progression, relative upper versus lower MN involvement, and in the occurrence of FTD. Even in families with a monogenetic cause of ALS, disease presentation is highly variable, suggesting that important disease-modifying factors exist (Regal et al., 2006). It is therefore clear that we need a better understanding of ALS disease mechanisms to identify potential therapeutic targets. Various disease models have already been developed to investigate ALS disease pathways. These models aim to recapitulate neuropathological or genetic aspects of the disease to uncover the molecular players involved in pathology that might be amenable to therapeutic intervention. Small-animal models, such as *Caenorhabditis elegans*, *Drosophila* and zebrafish can be used for unbiased forward genetic screens. Rodent models are considered to mimic human disease more closely and can be valuable for unravelling pathogenic mechanisms and for proof-of-concept studies. Induced pluripotent stem cells (iPSCs) and induced neurons (iNs) have also recently been used to model *in vitro* human neurodegenerative disorders. This reprogramming of somatic cells from patients with ALS is considered to be a valuable contribution to the researcher's toolbox (Matus et al., 2014).

Translation of findings from model systems to the clinic has been unsuccessful so far (Mitsumoto et al., 2014). Many of the therapeutic strategies were based on single study observations in rodent mutant SOD1 models and subsequently failed in a mostly sporadic ALS population. However, in recent years, our insights into the pathogenesis of MN degeneration have greatly improved. The heterogeneity in its genetic causes offers tremendous opportunities for creating novel disease models and for mechanistic studies. In this Review, we summarize the emerging new disease mechanisms identified using these different new model systems and discuss the most important translational opportunities and challenges.

**Box 1. Glossary of clinical terms**

**Bulbar**: refers to muscles innervated by the ‘bulbus’ or brainstem via the cranial nerves, i.e. muscles of the face, throat and tongue required for articulation, chewing and swallowing.

**Executive dysfunction**: disruption of the efficiency of executive functions, such as attention, planning, problem-solving and abstraction.

**Fasciculations**: spontaneous muscle twitches of individual motor units caused by uncontrolled discharges in motor axons.

**Frontotemporal dementia (FTD)**: dementia syndrome caused by degeneration of cortical neurons in the frontal and anterior temporal lobes. It can present with changes in behaviour or language dysfunction.

**Lower motor neurons**: motor neurons located in the brainstem nuclei and in the ventral horn of the spinal cord, which send their axons to the muscles. Clinical signs of lower motor neuron loss include: weakness, muscle wasting and fasciculations.

**Upper motor neurons**: motor neurons located in the motor cortex, sending commands to lower motor neurons. Clinical signs of upper motor neuron loss include: increased muscle tone, hyperreflexia and slowing of voluntary movements.

**Box 2. Glossary of terms linked to ALS pathophysiology**

**Astrogliosis**: proliferation and activation of astrocytes surrounding diseased motor neurons.

**Excitotoxicity**: neuronal toxicity induced by excessive stimulation of glutamate receptors. Motor neurons have been shown to be particularly vulnerable to AMPA receptor stimulation.

**Karyopherins**: a family of proteins involved in transporting molecules between the cytoplasm and the nucleus through nuclear pores.

**Microgliosis**: proliferation and activation of microglial cells surrounding diseased motor neurons.

**Ran-mediated nucleocytoplasmic transport**: GTP-binding nuclear protein Ran is a GTPase that is activated by a GTPase-activating protein (RanGAP), leading to the conversion of RanGTP to RanGDP. The conversion of RanGDP to RanGTP is mediated by RCC1, the nucleotide exchange factor for Ran. The nucleocytoplasmic gradient of Ran drives transport across the nucleocytoplasmic membrane through interactions with karyopherins loaded with cargo.

**Stress granules**: membrane-less cytoplasmic granules of mRNA and RNA-binding proteins formed upon stress to halt translation and to protect mRNAs from damage.

## Genes and pathways in ALS

Advances in our understanding of the genetics and neuropathology of ALS underpin much of our current knowledge of this disease. The discovery of mutations in *SOD1* in 1993 heralded the era of ALS disease modelling (Rosen et al., 1993). SOD1 is a cytosolic enzyme that catalyses the detoxification of superoxide, but mis-sense mutations in *SOD1* do not seem to cause ALS by a loss of dismutase activity. Instead, a toxic gain-of-function is thought to underlie the disease-associated role of this protein (Cleveland and Rothstein, 2001). In the following years, TDP-43 was identified as a major constituent of the ubiquitin-positive aggregates found in the MNs of ALS patients (as well as in the MNs of ∼50% of cases of FTD in the absence of ALS) (Neumann et al., 2006). In addition, disease-causing mutations were found in the TDP-43-encoding gene (*TARDBP*) (Kabashi et al., 2008; Sreedharan et al., 2008; Van Deerlin et al., 2008). Not long thereafter, mutations in *FUS* (Kwiatkowski et al., 2009; Vance et al., 2009) and *C9orf72* (DeJesus-Hernandez et al., 2011; Renton et al., 2011) were discovered. TDP-43 and FUS are RNA- and DNA-binding proteins that play a role in numerous cellular processes, including transcription, splicing, microRNA maturation, RNA transport and stress granule formation (Lagier-Tourenne and Cleveland, 2009). In line with their nuclear and cytoplasmic functions, TDP-43 and FUS can shuttle between the nucleus and the cytoplasm, but in basal conditions they are predominantly nuclear. The cytoplasmic mislocalization of TDP-43 and FUS in cytoplasmic protein aggregates and their subsequent nuclear depletion is an important hallmark of ALS. The nuclear loss of TDP-43 and FUS function, their cytoplasmic aggregation and aggregate-associated cytotoxicity are believed to contribute to ALS pathogenesis (Lagier-Tourenne and Cleveland, 2009; Liu et al., 2017).

For *C9orf72* mutations, which consist of an expanded GGGGCC hexanucleotide repeat in the first intron, three putative disease mechanisms have been suggested: reduced C9orf72 protein formation due to transcription interference caused by the repeat expansion; the formation of RNA foci by RNA that contains the repeat expansion, leading to cell toxicity (caused by their binding and depletion of RNA-binding proteins); and repeat-associated non-ATG (RAN) translation of sense and antisense repeat-containing RNA, which gives rise to aggregating dipeptide repeat (DPR) proteins (Ash et al., 2013; Mori et al., 2013). C9orf72 has also been recently implicated in the initiation of autophagy (Sellier et al., 2016; Webster et al., 2016), but how this relates to MN degeneration remains unclear.

In recent years, mutations in several other genes have been discovered in ALS, most of which are inherited in an autosomal dominant manner. Although many of these mutations are not frequently encountered, they hint at important disease pathways involved in MN vulnerability and degeneration (Fig. 1). Some of the genes function in protein degradation pathways, in line with an important role for impaired proteostasis in ALS. They include ubiquilin 2 (*UBQLN2*) (Deng et al., 2011), which links the ubiquination machinery to the proteasome, and also optineurin (*OPTN*) (Maruyama et al., 2010), TANK-binding kinase 1 (*TBK1*) (Cirulli et al., 2015; Freischmidt et al., 2015), valosin-containing protein (*VCP*) (Johnson et al., 2010) and p62/sequestosome 1 (*SQSTM1*) (Fecto et al., 2011; Rubino et al., 2012), which are important for cargo recognition and cargo delivery to the autophagosome. TBK1 can also phosphorylate OPTN and p62, further supporting their intrinsic connections (Richter et al., 2016). Other ALS-associated proteins affect vesicle transport and sorting, such as charged multivesicular body protein 2B (*CHMP2B*) (Skibinski et al., 2005), which functions in the sorting of endosomal ubiquinated cargoes into multivesicular bodies, and VAMP-associated protein B and C (*VAPB)* (Nishimura et al., 2004).

In addition to *TARDBP* and *FUS*, a number of other genes that encode RNA-binding proteins (RBPs) are mutated in ALS. Their encoded proteins function in RNA metabolism and in stress granule formation. For example, intermediate repeat lengths in ataxin 2 (*ATXN2*) (Elden et al., 2010; Van Damme et al., 2011) and mutations in heterogeneous nuclear ribonucleoprotein A1 and A2B1 (*hnRNPA1/A2B1*) (Kim et al., 2013) are linked to ALS. These RBPs have several features in common. They shuttle between the nucleus and cytoplasm, undergo liquid-liquid phase separation, fulfil important roles in the nucleus and are involved in cytoplasmic stress granule formation (Liu et al., 2017). Angiogenin (ANG), which has also been implicated in ALS, is a mediator of blood vessel formation and also functions as a ribonuclease that hydrolyzes cellular tRNAs (Gao and Xu, 2008).

Other genetic mutations associated with ALS underscore the importance of cytoskeletal organization and axonal transport for MN health. Mutations in genes encoding cytoskeletal proteins, such as tubulin α 4A (*TUBA4A*) (Smith et al., 2014), neurofilament heavy chain (*NEFH*) (Al-Chalabi et al., 1999) or profilin 1 (PFH1) (Wu et al., 2012), which is responsible for actin polymerization, suggest that the destabilization of axonal filaments is important. ALS-associated mutations in the dynactin subunit 1 gene (*DCTN1*) (Puls et al., 2003) also indicate the involvement of the axonal transport machinery in disease pathogenesis. Two recently discovered ALS-associated genes, chromosome 21 open reading frame 2 (*C21orf2*) (van Rheenen et al., 2016) and NIMA-related kinase 1 (*NEK1*) (Kenna et al., 2016) are involved in cilia formation, but also interact with each other in a complex involved in DNA repair. It is not yet known how these genes contribute to ALS pathogenesis.

The many genetic mutations that are currently associated with ALS and that are found in ALS patients with varying prevalence, indicate that the biological pathways that can contribute to MN degeneration are more heterogeneous than previously anticipated. Disease models for many of the recently discovered genes are underway and are expected to shed light on the respective disease mechanisms in the different genetic subtypes of ALS. Hopefully, this will also reveal common pathways relevant to all forms of ALS.

## Models of ALS

To identify novel therapeutic targets for ALS, it is essential to have a much better understanding of the disease mechanisms that lead to MN degeneration. To investigate these mechanisms, researchers are modelling certain aspects of ALS in a range of model systems, guided by recent insights into its genetic and molecular basis, to shed light on the complex process of MN degeneration. Such model systems vary from *in vitro* biochemical systems, to cell culture systems, invertebrates and non-mammalian vertebrates, and extend to rodent models, and more recently, to human patient-derived stem cell models.

**Fig. 2.**
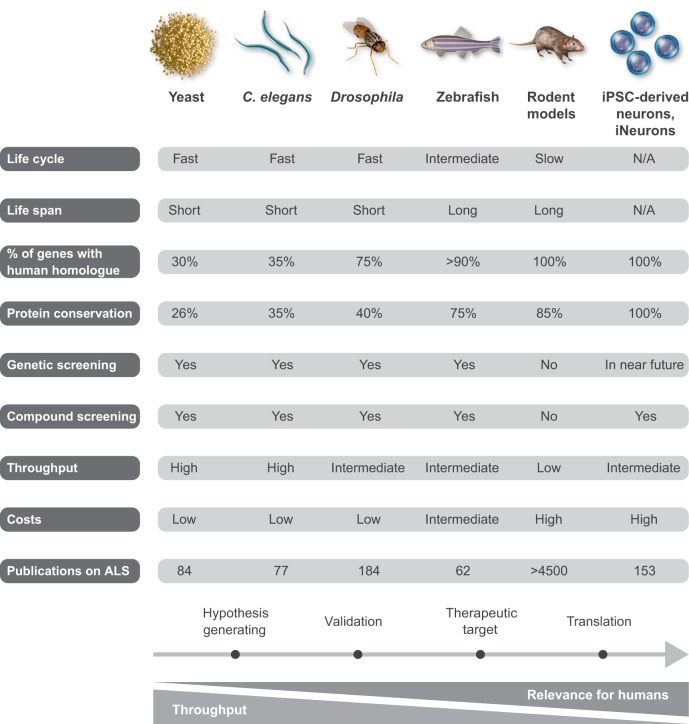
**Model systems in ALS research.** A summary of the advantages and limitations of the different model systems – yeast, *C. elegans*, *Drosophila*, zebrafish, rodents and iPSC-derived neurons – currently used to study ALS.

Each of these model systems has its advantages, as well as its limitations, as summarized in Fig. 2. In recent years, the availability of patient-derived stem cell models (Matus et al., 2014), has opened up new avenues for ALS research and provides a good balance between throughput and relevance for human disease. However, the translational value of each of the model systems remains unclear, as examples of successful translation to patients are still lacking. Yet, it remains possible that a cross-model approach, in which novel disease mechanisms identified in less-complex systems and later validated in more-complex models as well as in human cells or samples, have a higher chance of successful translation to the clinic. Below, we discuss the progress and possibilities of ALS disease models and provide examples of important insights based on these models. Table 1 gives an overview of existing model systems for the different genetic subtypes of ALS.

**Table 1.**
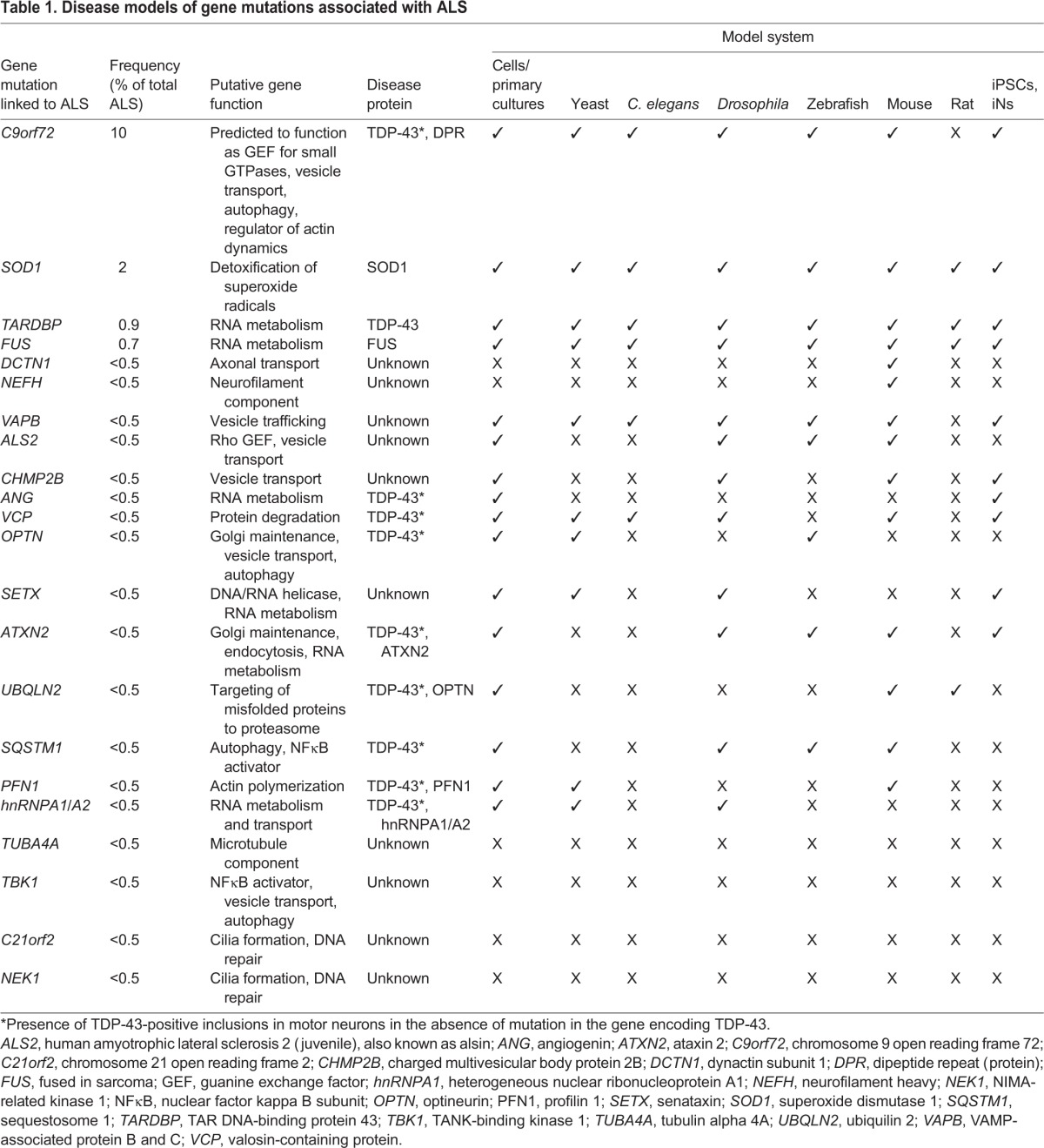
**Disease models of gene mutations associated with ALS**

### Cellular systems

As discussed earlier, many of the genetic mutations associated with ALS lead to the production of aberrant proteins. A common feature of these ALS disease-associated proteins is their propensity to misfold and aggregate. *In vitro* oligomerization and aggregation assays help us to understand the biophysical principles of protein aggregation. Many of the ALS-associated proteins contain prion-like domains, which promote aggregation (Boeynaems et al., 2016b). More recently, low-complexity or intrinsically disordered domains, which are present in many ALS-associated proteins, have been found to mediate the process of liquid-liquid phase separation, whereby proteins separate from a watery solution and migrate into liquid-like droplets (Han et al., 2012; Molliex et al., 2015).

Various cell lines are used to study ALS-associated gene function or the toxicity related to the overexpression of wild-type or mutant proteins. Although such simple disease models do not recapitulate the complexity of human disease, they can help to uncover important information concerning the biological function of ALS-associated genes and proteins. Such studies have shown, for example, the importance of low-complexity domains in ALS proteins for stress granule formation (Molliex et al., 2015) and the role of C9orf72 in autophagy (Webster et al., 2016).

One cellular model that is particularly suited for modelling cytotoxicity induced by ALS proteins is the budding yeast *Saccharomyces cerevisiae*. A simple growth assay reveals that many ALS-associated proteins cause reduced growth, which can be used for phenotypic screening (Elden et al., 2010; Jovičić et al., 2015; Kim et al., 2014). Various genetic tools are available for genetic screens, including libraries to perform overexpression or single-gene deletion screens. About 30% of yeast genes have a human homologue and several genes identified in yeast genetic screens have been successfully validated in higher-order models. The RNA-binding protein ATXN2 has been identified in yeast as a modifier of TDP-43 toxicity, and as an interactor of TDP-43. This led to the discovery of intermediate repeat expansions in *ATXN2* as a genetic risk factor for human ALS (Elden et al., 2010). ATXN2 is recruited to stress granules, as are TDP-43 and FUS. Other stress granule-localized proteins were found to modify TDP-43 toxicity in yeast (Kim et al., 2014). This finding introduced the concept that stress granule dysfunction is involved in the pathogenesis of ALS. Another recent discovery that emerged from genetic screens in yeast is the involvement of the nucleocytoplasmic transport machinery in mediating the cytotoxicity induced by dipeptide repeat proteins (Jovičić et al., 2015). The proteins involved include several karyopherins (Box 2) and effectors of Ran-mediated nucleocytoplasmic transport (Box 2). Pathogenic mutations have not been identified in any of these modifiers, to date. Primary cultures of rat or mouse MNs have often been used to investigate MN vulnerability to a range of factors, including glutamate (Van Damme et al., 2007) and toxic astrocytic factors released from ALS patients or in rodent models of ALS (Nagai et al., 2007; Song et al., 2016). More recently, primary neuronal cultures have been used to investigate the neurotoxicity of the dipeptide repeat proteins that are produced by RAN translation in patients with GGGGCC repeat expansions in *C9orf72* (Wen et al., 2014). These studies found that MNs are selectively vulnerable to certain insults and that several compounds and neurotrophic factors can enhance MN survival *in vitro* (e.g. Bordet et al., 2007). Although these cellular models only remotely mimic the disease, their merit lies in their potential to study the role of genes involved in ALS, to assess the consequences of gene mutations, to model protein aggregation and to identify disease-modifying genes or compounds in unbiased screens.

### Small-animal models

A key limitation of cellular models is that they lack the complex interplay that occurs between MNs and their surrounding environment in a living organism. Although rodent models are considered the gold standard for validating disease mechanisms and for providing preclinical data on potential therapeutic targets, small-animal models are increasingly being used to model the diverse genetic causes of ALS. They can be generated quickly, are cheap to maintain relative to rodent models and are amenable to genetic or compound screening. In addition, interactions between different genetic causes and between different genetic modifiers can be studied in whole animals in order to identify the genetic networks involved in neurodegeneration.

### Drosophila melanogaster

By far the most widely used small-animal model of ALS is the fruit fly, *Drosophila melanogaster*. The fruit fly is an attractive disease model for several reasons. It has ∼14,000 genes on 4 chromosomes, and about 75% of human genes have a functional orthologue in the fly with, on average, about 40% homology (McGurk et al., 2015). The fly has a very rapid life cycle and its lifespan is only ∼50 days. The nervous system is also quite sophisticated and contains approximately 100,000 neurons (Pandey and Nichols, 2011), and there are a number of tools and assays available to study its function (Ugur et al., 2016).

The repertoire of genetic tools available for *Drosophila* is extensive: transgenic flies that overexpress a transgene of interest or a small interfering RNA can be rapidly generated, and the GAL4/UAS system allows for cell-specific expression or gene knockdown. Moreover, the tools for genetic screens, created by crossing wild-type flies to deletion stocks or to RNAi lines (which cover ∼90% of the genome and are publicly available), and for chemical mutagenesis are extremely efficient (Ugur et al., 2016). Fly models overexpressing mutant human transgenes for ALS linked to mutations in *SOD1* (Watson et al., 2008), *TARDBP* (Estes et al., 2011; Feiguin et al., 2009; Vanden Broeck et al., 2013), *FUS* (Chen et al., 2011; Lanson et al., 2011), *C9orf72* (Mizielinska et al., 2014; Xu et al., 2013), *ALS2* (Takayama et al., 2014), *VAPB* (Ratnaparkhi et al., 2008; Tsuda et al., 2008), *VCP* (Johnson et al., 2015) and *SETX* (Mushtaq et al., 2016) exist. These transgenic models for ALS show that the overexpression of the wild-type or mutant version of each of these proteins induces cellular toxicity. For *TARDBP* and *FUS*, both gene deficiency and overexpression were toxic (Lanson et al., 2011; Vanden Broeck et al., 2013). This suggests that TDP-43 and FUS are essential proteins, with tightly regulated levels, and that minor changes in their levels or protein sequence has far-reaching consequences for neuronal health. Neuronal toxicity induced by expanded GGGGCC-repeat RNA (Xu et al., 2013) and by the direct expression of codon-optimized dipeptide repeat proteins (Mizielinska et al., 2014) has also been shown in *Drosophila*. In particular, the neuronal toxicity of the arginine-containing dipeptide repeat proteins, proline-arginine (PR) and glycine-arginine (GR), has been modelled in the fly (Mizielinska et al., 2014; Tran et al., 2015; Wen et al., 2014). The transcriptional activator protein Pur-α is a binding partner of the expanded GGGGCC RNA, and its overexpression rescued the neuronal toxicity induced by this repeat RNA in the fly (Xu et al., 2013). Together with other RBPs, Pur-α is present in RNA foci and stress granules; it also regulates stress granule dynamics and counteracts motor neuron death induced by overexpression of mutant FUS in primary MNs overexpressing mutant FUS (Daigle et al., 2016).

Targeted genetic screens and validation experiments in *Drosophila* have also been instrumental to uncover several ALS disease mechanisms. *ATXN2* was confirmed to be a modifier of TDP-43 toxicity in the fly, as its overexpression enhanced the retinal toxicity induced by TDP-43, while its downregulation mitigated toxicity (Elden et al., 2010). Targeted genetic screens in *Drosophila* have also identified several important players in nucleocytoplasmic transport as modifiers of the cytotoxicity induced by GGGGGCC-repeat RNA or by dipeptide repeat proteins (Boeynaems et al., 2016a; Freibaum et al., 2015; Zhang et al., 2015). Experiments in yeast first pointed to a role for stress granule dysfunction in ALS. However, an important role for poly-(A)-binding protein (PABP) and eIF2a phosphorylation (both involved in stress granule formation) as modifiers of TDP-43 toxicity was later confirmed in *Drosophila* (Kim et al., 2014).

### Caenorhabditis elegans

The nematode *C. elegans* is also being used to study the genetic pathways that contribute to ALS (Therrien and Parker, 2014). *C. elegans* has an extremely short life cycle of only 4 days and is easy to manipulate genetically. About 35% of human genes have worm orthologues, typically with about 30% protein sequence homology (Therrien and Parker, 2014). The nervous system of this nematode is simple but very well characterized, with all 302 neurons and their connections carefully mapped (Therrien and Parker, 2014). GFP fusion proteins can make specific proteins visible *in vivo* over time, because of the transparency of *C. elegans*. Moreover, many mammalian cellular stress and survival pathways are conserved in the worm. *C. elegans* models of the four main ALS-associated genetic mutations (*SOD1*, *C9orf72*, *TARDBP* and *FUS*) have been created (Therrien and Parker, 2014) and have contributed to several important recent insights. For example, calcineurin was identified in a *C. elegans* model of TDP-43 proteinopathy as an important phosphatase involved in the removal of pathological C-terminal phosphorylation of TDP-43 (Liachko et al., 2016). Another *C. elegans* ALS model revealed that the N-terminal low-complexity domain of FUS (which mediates the liquid-liquid phase separation that allows FUS to enter liquid-like membrane-free granules, such as stress granules) is necessary for neuronal toxicity caused by aberrant FUS (Murakami et al., 2015). Evidence for ER stress and disturbed Ca^2+^ homeostasis as modifiers of MN degeneration was generated using *C. elegans* models expressing human mutant TDP-43 or SOD1 (Aggad et al., 2014; Jablonski et al., 2015). Interestingly, modifying the expression levels of the ALS risk-enhancing genes *UNC13A* (van Es et al., 2009) and *SARM1* (van Rheenen et al., 2016) also modified neurodegeneration in *C. elegans* models of mutant TDP-43 and FUS (Veriepe et al., 2015). Moreover, knockdown of the orthologue of dynactin 1 in *C. elegans* resulted in a motor phenotype that indicates a potential role for autophagosome transport in MN maintenance (Ikenaka et al., 2013).

### Zebrafish

Zebrafish (*Danio rerio*), often used to study embryonic development because of the transparency of their embryos and their vertebrate body plan (Phillips and Westerfield, 2014), are increasingly being used in ALS research because of the advantages this model offers (see Fig. 2) (Patten et al., 2014). Most human genes have a zebrafish homologue, typically with about 70% homology in protein sequence (Phillips and Westerfield, 2014). Zebrafish can be used for compound screening. In addition, gene overexpression or knockdown can be easily achieved using injections of RNA (or cDNA) or morpholinos (a form of antisense RNA) at the 2- to 4-cell embryonic stage. More recently, gene-deletion strategies (such as CRISPR/Cas9) have been used to combat the off-target effects of some morpholinos (Gerety and Wilkinson, 2011). Stable transgenic zebrafish have been generated for in-depth studies of disease pathogenesis in ageing fish (with a life span of up to 2 years) (Ramesh et al., 2010).

In ALS research, transient embryonic models have been used to model the toxic effects of ALS-associated genetic mutations. These models, which were generated by injecting cDNA or RNA in fertilized eggs, have provided evidence that mutant SOD1 leads to neuronal toxicity via a gain-of-function (Sakowski et al., 2012; Van Hoecke et al., 2012). The same models have been used to demonstrate combined gain- and loss-of-function mechanisms for *TARDBP*, *FUS* and *C9orf72* (Armstrong and Drapeau, 2013; Ciura et al., 2013; Hewamadduma et al., 2013; Lee et al., 2013; Schmid et al., 2013), as both reducing and increasing gene expression levels induced motor neuron toxicity.

Additionally, interactions between different ALS-associated genes can be readily studied in zebrafish. Zebrafish experiments have revealed commonalities between TDP-43- and FUS-induced MN toxicity. For example, wild-type TDP-43 and FUS could rescue MN toxicity induced by knockdown of the respective genes, but not the toxicity induced by mutant SOD1 (Kabashi et al., 2011). Furthermore, the neurotrophic factor progranulin was found to improve the MN toxicity induced by mutant TDP-43 and FUS, but not of mutant SOD1 (Kabashi et al., 2011; Laird et al., 2010). A zebrafish morpholino-based modifier screen identified the ephrin receptor EphA4 as a modifier of ALS (Van Hoecke et al., 2012). The authors then confirmed that lowering EphA4 levels has a beneficial effect on disease progression in mutant SOD1 mice and that low EphA4 levels are associated with milder clinical manifestations in ALS patients (Van Hoecke et al., 2012). In mutant SOD1 zebrafish, the earliest evidence of pathogenicity is interneuron dysfunction resulting in reduced inhibitory input to spinal motor neurons. In a compound screening, riluzole and apomorphine (an activator of the regulator of resistance to oxidative stress, nuclear factor erythroid 2-related factor 2 or NRF2) counteracted this interneuron dysfunction (McGown et al., 2013). Stimulation of resistance to oxidative stress by NRF2 overexpression in astrocytes also prolonged survival in mutant SOD1 mice (Vargas et al., 2008), suggesting that this pathway warrants further study in ALS.

Although the small-animal models discussed here have not yet given rise to new therapies in patients, they play an increasingly important role in ALS research. Owing to the speed and ease with which transgenic lines can be generated, disease models for each of the genetic subtypes of ALS can readily be made to facilitate in-depth study of the interactions between the different forms. Thanks to improved genetic engineering technologies, there is a tendency to use fewer overexpression models and to switch to knock-in models. In addition, unbiased genetic screens are feasible and can reveal key disease modifiers. Compound screening is also possible, especially in *C. elegans* and zebrafish. For all these reasons, these small-animal models are particularly suited as hypothesis-generating platforms that can reveal novel potential therapeutic targets ready for validation in complex but low-throughput rodent models.

### Rodent models of ALS

Rodent models exist for genetic mutations that are most prevalent in ALS patients and are widely used to study disease mechanisms. An in-depth discussion of all recent ALS rodent models is beyond the scope of this article, and we refer readers to recent reviews on rodent models for ALS-associated mutations in *SOD1*, *TARDBP*, *FUS* and *C9orf72* for more information (Gendron and Petrucelli, 2011; Nolan et al., 2016; Philips and Rothstein, 2015; Turner and Talbot, 2008). Transgenic mice and rats represent the gold standard of preclinical ALS modelling and these animal models have provided important insights into MN degeneration pathways (Ferraiuolo et al., 2011). In particular, mouse models in which human genomic mutant *SOD1* is overexpressed have been used intensively since the first of such models became available in 1994 (Gurney et al., 1994) and much of our current understanding of disease mechanisms is based on this model. Although overexpression of the transgene is an important limitation of these models, the significant correlation between the expression level of mutant SOD1 and the severity of MN degeneration (Alexander et al., 2004) paved the way for gene-silencing strategies using antisense oligonucleotides (ASOs) to treat mutant SOD1-mediated ALS (Smith et al., 2006; van Zundert and Brown, 2017).

Protein misfolding and aggregation is observed prior to MN death in rodent models, as in ALS patients (Chattopadhyay and Valentine, 2009). A prion-like, cell-to-cell migration of misfolded SOD1, which is secreted by MNs (Urushitani et al., 2006) and taken up by neighbouring cells, is an emerging concept that has potentially important therapeutic implications. Defects in protein-clearance mechanisms might also underlie problems with protein turnover (Ruegsegger and Saxena, 2016). Apart from overloading the ubiquitin-proteasome system, alterations in autophagosome formation, in vesicle transport and in lysosome fusion, to induce the degradation of damaged proteins and organelles, have been implicated in ALS (Cipolat Mis et al., 2016). Preclinical studies in mutant *SOD1* mice suggest that boosting autophagy could slow down disease progression (Cipolat Mis et al., 2016; Staats et al., 2013).

A neuroinflammatory response is common to all forms of ALS and most likely modulates the process of neurodegeneration (Philips and Robberecht, 2011). Cell type-specific transgenic mouse models, using the Cre-Lox system to excise the mutant *SOD1* transgene from specific cell-types, have greatly advanced our understanding of the importance of non-neuronal cell types in MN degeneration, such as microglia (Boillee et al., 2006), astrocytes (Yamanaka et al., 2008) and oligodendrocytes (Kang et al., 2013). The importance of α-amino-3-hydroxy-5-methyl-4-isoxazolepropionic acid (AMPA) receptor-mediated excitotoxicity in MN death has been shown in rodent models of ALS (Van Damme et al., 2005b), with a loss of the astrocytic glutamate transporter EAAT2 (Rothstein et al., 2005) and altered AMPA receptor subunit composition (Van Damme et al., 2007; Van Damme et al., 2005a) contributing to increased extracellular glutamate levels and to MN vulnerability to glutamate stimulation, respectively.

Alterations in mitochondrial morphology, function, clearance and transport have also been observed in mutant SOD1 mice, and this can lead to energy failure and to oxidative stress (Shi et al., 2010). Moreover, inhibition of the mitochondrial permeability transition-mediated cytochrome c release, using minocycline, resulted in a significantly longer survival of mutant SOD1 mice (Zhu et al., 2002), an effect that was confirmed by others (Kriz et al., 2002; Van Den Bosch et al., 2002). A subsequent trial with minocycline in patients with ALS failed (Gordon et al., 2007); however, the drug has not yet been tested in a population of ALS patients with *SOD1* mutations.

Studies in mutant SOD1 mice have revealed that the disease process starts with a dying back of axons (axonopathy), characterized by axonal retraction from neuromuscular junctions and by axonal degeneration prior to the loss of cell bodies in the ventral horn of the spinal cord (Fischer et al., 2004). Alterations in cytoskeletal organization, axonal transport of mitochondria, RNA, vesicles and other cargoes, and changes in axonal outgrowth cues (such as EphA4 and Nogo-A) might all contribute to this axonal degeneration (Peters et al., 2015). In line with this idea, the protection of neuronal survival, either by deleting the pro-apoptotic *Bax* gene or by inhibiting p38 MAPK, is insufficient to extend dramatically the survival of mutant SOD1 mice (Dewil et al., 2007; Gould et al., 2006).

The mutant SOD1 rodent models have also contributed to the concept that neurotrophic factors play a crucial role in MN degeneration. One example of this is vascular endothelial growth factor (VEGF). A transgenic mouse model in which the ‘hypoxia response element’ in the promoter region of the *Vegf* gene was deleted developed an ALS-like phenotype (Oosthuyse et al., 2001). In addition, *Vegf* expression was lower in the spinal cords of mutant SOD1 mice before disease onset (Lu et al., 2007). The phenotype of these mice improved by overexpressing VEGF; additionally, the survival of the mutant SOD1 rat model increased on VEGF treatment. These findings suggest that VEGF contributes to and could modify neurodegeneration in ALS (Storkebaum et al., 2005; Wang et al., 2016).

Rodent models remain the gold standard ALS model, because they are a mammalian model system with a high degree of genetic homology to humans, enabling detailed mechanistic studies with palpable relevance to human physiology. However, they are much more expensive to maintain and time-consuming to generate compared with small-animal models and do not allow for testing multiple hypotheses at the same time. Another limitation is the inbred nature of mouse colonies, which does not reflect the human situation and can give rise to variable results when crossbreeding with other transgenic lines is required. Most mouse models are still overexpression models, but by using novel genetic engineering technologies, knock-in models will become more readily available.

### Stem cell models

The ability to generate induced pluripotent stem cells (iPSCs) from differentiated cells has opened up new avenues for ALS research. By introducing pluripotency genes into human patient-derived cells, it is now feasible to generate iPSCs from patients with ALS (with or without known disease-causing gene mutations) (Matus et al., 2014). In addition, iPSCs can be differentiated into spinal motor neurons (Maury et al., 2015) to study the specific cell type of interest in ALS. Direct conversion of fibroblasts into neurons has become feasible as well (Son et al., 2011). Although the translational potential of these ALS models remains to be proven, they seem to have many advantages and a good balance between throughput and relevance for the human disease (Matus et al., 2014). One of the most important advantages of generating models from patient-derived stem cells is that they remove the need to overexpress transgenes containing pathogenic ALS gene mutations. In addition, they carry endogenous gene mutations in the context of an individual patient's genetic background. Sporadic ALS can also be modelled using this approach, which is not possible in the other model systems.

The protocols for generating spinal motor neurons are improving, but the iPSC models could be of greater value if robust systems were available to generate mature MNs that innervate muscle cells (Sances et al., 2016). Generating upper motor neurons also remains a challenge (Sances et al., 2016). Using gene-editing techniques, such as CRISPR/Cas9, it is possible to correct disease-causing mutations in stem cell models and to compare patient lines to their corrected counterpart – an important advantage of this approach, given the considerable biological variability between different lines (Kiskinis et al., 2014; Sances et al., 2016). Many of the first ALS iPSC models have revealed interesting *in vitro* phenotypes (Matus et al., 2014), which are valuable for mechanistic studies and for designing novel therapeutic strategies. Several aspects of the disease's neuropathology are recapitulated in iPSC-derived motor neurons, such as the aggregation and/or cytoplasmic mislocalization of TDP-43, SOD1, FUS, and in the case of C9orf72 repeat expansions, the formation of RNA foci and dipeptide repeat proteins (Matus et al., 2014). Many important functional phenotypes have already been identified in iPSC-derived spinal motor neurons, ranging from increased cell death (Bilican et al., 2012), neurofilamentous disorganization (Chen et al., 2014), defects in nucleocytoplasmic transport (Zhang et al., 2015), to changes in excitability (Kiskinis et al., 2014). An example of the latter is provided by a study using MNs derived from patients with an A4V mutation in *SOD1*. These MNs displayed reduced delayed-rectifier potassium currents, giving rise to hyperexcitability; boosting the currents with retigabine blocked the hyperexcitability and improved MN survival *in vitro* (Wainger et al., 2014). Patient-derived cells have also been used to confirm the importance of non-neuronal cells in human ALS models without overexpression of mutant transgenes (Ferraiuolo et al., 2016; Meyer et al., 2014). In addition, patient-derived MNs have proved to be invaluable to test therapeutic interventions, such as the use of ASOs (Lagier-Tourenne et al., 2013; Sareen et al., 2013) and to perform compound screening (Egawa et al., 2012). Genetic screens using CRISPR/Cas9 technology on phenotypic read-outs in iPSC-derived MNs will hopefully be possible in the near future (Qi et al., 2017).

Expectations are high for patient-derived stem cell models. They contain the genetic make-up of an individual who has developed ALS, even when the actual cause of the disease is unknown. Ideally, a component of ageing should be built into these models as well as the complex interactions with surrounding non-neuronal cells.

## Conclusions

The hitherto unsuccessful translation of findings from model organisms to patients can be accounted for both by factors related to the model systems and by factors related to the trial design in patients (Mitsumoto et al., 2014). The heterogeneity in ALS causes has been underestimated in the past and may have contributed to a targeting of the wrong patient populations. In mice, treatments are often started prior to disease onset, whereas patients are usually well into their disease course before entering a trial. Yet, the most important obstacle to develop new therapies is arguably our failure to grasp the cause of the disease in the majority of the patients.

Nonetheless, important progress has been made in our understanding of the genetics and neuropathology of ALS, opening up new avenues for disease modelling research and for better characterizing the disease pathways that contribute to ALS. Many different models are now being generated to investigate the recently identified ALS-associated genes, several of which seem to cluster into functional networks. Amongst others, these include RNA metabolism, protein degradation, vesicle transport, autophagy, cytoskeletal organization and axonal transport. iPSC-derived MNs expand the repertoire of ALS disease models and offer new opportunities, particularly for modelling sporadic ALS. Many fascinating disease mechanisms are emerging from models of ALS, such as impairments in nucleocytoplasmic transport, alterations in stress granule dynamics, transcriptional dysregulation, and non-neuronal cells acting as modulators of the disease (Taylor et al., 2016). Our progress in the availability of multiple disease models for ALS research and in the knowledge about the biology of the disease offers hope that robust therapeutic targets will be identified in the near future.
